# Smartphone-based Respondent Driven Sampling (RDS): A methodological advance in surveying small or ‘hard-to-reach’ populations

**DOI:** 10.1371/journal.pone.0270673

**Published:** 2022-07-21

**Authors:** Filip Lukasz Sosenko, Glen Bramley

**Affiliations:** 1 The University of Glasgow, Edinburgh, United Kingdom; 2 Heriot-Watt University, Edinburgh, United Kingdom; Iran University of Medical Sciences, ISLAMIC REPUBLIC OF IRAN

## Abstract

Producing statistically robust profiles of small or ‘hard-to-reach’ populations has always been a challenge for researchers. Since surveying the wider population in order to capture a large enough sample of cases is usually too costly or impractical, researchers have been opting for ‘snowballing’ or ‘time-location sampling’. The former does not allow for claims to representativeness, and the latter struggles with under-coverage and estimating confidence intervals. Respondent Driven Sampling (RDS) is a method that combines snowballing sampling with an analytical algorithm that corrects for biases that arise in snowballing. For all its advantages, a major weakness of RDS has been around data collection. Traditionally done on-site, the process is costly and lengthy. When done online, it is cheaper and faster but under a serious threat from fraud, compromising data quality and validity of findings. This paper describes a real-life application of a RDS data collection system that maximizes fraud prevention while still benefiting from low cost and speedy data collection.

## Introduction

Producing statistically robust profiles of small populations has always been a challenge for social researchers [1]. The task is even more elusive if the population under study is not only small but also ‘hard to reach’, meaning that its members may be less willing (or less able) to respond to the survey than those in the general population. In theory, the issue of small population size can be addressed: taking a sufficiently large sample of the general population would yield enough responses from the population of interest to arrive at a statistically robust profile of it. But that is very costly and does not address the issue of ‘hard-to-reach-ness’.

For a long time, the problem seemed intractable. The choice was really between recruiting survey respondents through opportunistic ‘snowballing’, or at services used by members of the population (‘time-location sampling’ [2]). These approaches allow for obtaining a desired sample size, but at the same time entail losing the statistical benefit of having a probabilistic sample. In snowball sampling, various biases occur: men are more likely to recruit peers that are male, people who are well networked are more likely to recruit more peers than those who are socially isolated, etc [3]. Time-location sampling, in turn, misses those who do not use services (under-coverage) and is skewed towards those who use them frequently [4]. In snowballing there is no way of estimating how confident one could be that the results are close to true population characteristics, while in time-location sampling there is no consensus on the extent to which such estimation can be done, and how (e.g. [2, 4]).

Another option has been available since the late 1990’s, however. Respondent Driven Sampling (RDS), developed by Douglas Heckathorn in the United States, combines snowballing sampling with an analytical algorithm that corrects for biases that arise in this very snowballing [3]. When the authors embarked on a research project aiming to arrive at a statistically robust socio-demographic profile of Polish migrants living in Luton (a satellite town of Greater London), we decided to employ RDS (see [5] for the study report). Being aware of the danger of survey fraud when data is collected online and participants are incentivised financially, we designed a way of collecting RDS data that maximises fraud prevention.

The study has been approved in writing by the Ethics Committee of the School of Energy, Geosciences, Infrastructure and Society, Heriot-Watt University. Participants’ consent was informed and obtained through ticking a checkbox in the online survey questionnaire. The study did not involve minors.

## What is RDS?

RDS is a three-step research method, with unique features in each step [6]. It is crucial that all three steps are conducted as prescribed in the RDS ‘rulebook’. Firstly, in the design (‘formative assessment’) step, the researcher must carefully think through what the target population is and whether the study design is likely to meet RDS assumptions. Of particular importance is the assumption that the population of interest is homogenous, in the sense that personal networks within it do not cluster along some characteristics, for example language, ethnicity or class. The presence of such internal ‘population splitters’ would mean that snowballing is likely to be affected by them; the resulting sample would probably be not representative of the overall target population, and the RDS algorithm would be unlikely to sufficiently correct for this. Beside this, the researcher must calculate the required sample size (conditional on confidence intervals that the researcher is comfortable with), or what confidence intervals are going to be (conditional on a pre-determined sample size).

In the second step—sampling and data collection—‘seed’ respondents are recruited by the researcher. Typically, their number varies between 5–15. Each ‘seed’ is assigned a unique identifier and asked to fill in the survey questionnaire with questions substantive for the study. Additionally, the survey must include a question about the size of the personal network (in the target population). Practically, this is elicited via a question such as ‘In the last seven days, how many people who are Polish, living in Luton and aged 18 or over did you meet in person or via phone / text / social media? You don’t need to know them well, it’s enough if you know their name and they know yours’. This information is later used by the analytical RDS algorithm to estimate the probability of the respondent to be selected for the survey. Once the ‘seed’ has filled in the survey questionnaire, he/she is given a ‘thank you’ reward (usually financial) and a few (typically 2–3) unique invitation codes to distribute between friends / acquaintances / family in the target population. Those follow-up contacts who end up being recruited fill in the survey questionnaire, receive their ‘thank you’ reward and further invitation codes. Those who recruited them receive a secondary reward, usually smaller than the primary reward (in our case the primary reward was £10 and the secondary was £5). Data collection continues until the target sample size is reached.

Lastly, in the data analysis step the researcher applies one of the existing RDS algorithms to analyse the data. Currently it is recommended to use a third generation of RDS algorithms, the Gile’s Sequential Sampling estimator [7]. The estimator corrects for biases that occur in snowballing–notably ‘homophily’, that is a tendency to recruit people similar to us–and allows for estimation of confidence intervals. At the time of writing the Gile-SS estimator was available as an R package and as a stand-alone, free software RDS-A. Importantly, recent work on RDS estimators suggests that design effects in RDS are of similar magnitude to those in typical complex survey sampling [7].

## RDS data collection process

Most RDS studies collect data through face-to-face contact (e.g. [8–10]). The research team would set up an office in a location that is convenient for the population under study to get to, and participants would need to come to that study office to fill in the survey and receive their rewards. This allows—to a smaller or larger extent—for verification that the participant belongs to the target population (for example place of residence or sex). The disadvantages are that it is costly and takes a long time to reach the target sample size, sometimes over one year. Also importantly, getting to the office requires effort from participants, creating a real risk of response bias: people who are better-off or who live far from the office are less incentivised than people who are on low income or who live close to the study office.

In the last decade however, more and more RDS researchers have been collecting data via phone and/or online [e.g. [11]]. This reduces costs and speeds up the process but creates new challenges around data quality, due to the possibility of fraud. If the respondent is interviewed over the phone, it is usually not possible to check if he/she lives in the study area, or to verify characteristics such as age or ethnicity. To some extent the location issue is tackled if the reward is later posted to a physical address, but the fraudster can give a friend’s address. Collecting data through online surveys, in turn, opens up a Pandora’s Box, as described in the next section.

## The two key challenges with online RDS

### 1. Online survey fraud

This challenge is faced by any research that uses online surveys with incentives, not just the RDS. The body of evidence on online survey fraud has grown in the past decade, but the subject is still relatively under-researched [12–14]. What is known currently points at two kinds of fraudulent behaviour that compromises data quality:

#### (A) Multiple participation

A participant may try to obtain more than one incentive by filling in the survey more than once. Existing evidence suggests that this used to be infrequent in early days of online surveys but has gradually increased in prevalence [12]. The impact on data quality depends on how many times a participant filled in the questionnaire and whether the responses were truthful or not. The spectrum ranges from two submissions from one individual, each with truthful answers, to dozens or more of submissions with random answers.

In the case of the RDS, the problem takes the form of a participant trying to fill in the survey more than once using the same invitation code or trying to use a follow-up invitation code that is supposed to be used by a contact, masquerading as a ‘child’ respondent.

The main ways of preventing this kind of fraud have been via the use of cookies, IP address, or email address [12, 15]. All three ways are imperfect: most people have more than one email address or can easily generate a new one, which makes it easy to participate more than once; IP address is dynamic on mobile devices and in some countries (e.g. in EU countries) cannot be legally obtained without the prior consent; and cookies can be cleared (this requires some level of technological know-how though, so using cookies is probably the strongest of these three solutions).

#### (B) Ineligible participation

This issue is a flip coin side of what is often seen as an advantage of online data collection, respondent anonymity. Three types of problems may occur:

1. The respondent may try to respond to the survey despite not being invited to the study. In the RDS, this may take the form of a fraudster trying to guess the invitation code or trying to use someone else’s invitation code.
2. The respondent may not meet the requirement of residing in the area under study. In RDS research, which often takes place in a well-defined location, this criterion is very important.
3. The fraudulent respondent may not meet one or more of the demographic eligibility criteria, such as being male or aged 18+, and give false answers to relevant survey questions. This kind of fraud is difficult to prevent or detect. Later in the article we offer a suggestion for tackling this problem.

Multiple participation and ineligible participation are not exclusive. Hence, in the worst-case scenario, data quality may be compromised by multiple entries from an ineligible individual.

It needs to be mentioned that traditional, on-site RDS is also not immune to fraud. The practice of ‘trading’ follow-up invitation codes, i.e. selling them to someone outside of one’s social network, has been of particular concern to RDS researchers [9].

Unfortunately, RDS researchers using online data collection tend to ignore or gloss over the issue of fraud. For example, in Wejnert & Heckathorn [11] coupons were distributed via email but the authors do not discuss the possibility of fraud through multiple participation. Bengtsson et al. [16] discuss the ways in which multiple participation was prevented in their study but not remaining ways in which the system could have been exploited. Tucker et al. [17] only state that ‘Chain development was checked regularly to identify duplicate or fake enrollment attempts, which were uncommon’. In contrast, we see online survey fraud as a serious and likely scenario, and we not only describe fraud attempts prevented by our system but also those that could not be prevented.

### 2. Poor quality of responses from legitimate participants

In a typical non-online survey, the interview is conducted face-to-face or over the telephone. The trained interviewer determines the pace of the interview and reads out questions clearly. A second serious data quality issue for online surveys (not specifically RDS) is to do with the possibility that the respondent may rush through the questionnaire, with little attention or even without reading questions and response options [12]. (This is probably more likely to happen when there is an incentive to claim: if there is none, the respondent is probably taking part because he/she sees the value in providing a response). This issue has been raised particularly in relation to ‘professional survey takers’ [18], but clearly can happen with other respondents too. A range of solutions have been proposed, including offering a low incentive or a prize draw, discarding submissions with a suspiciously short response time, or a sensitivity analysis with and without the suspect submissions [12].

Of these proposals, we would argue that solutions relying on timing of responses are preferred in the context of RDS. A prize draw is unlikely to generate enough interest in the survey and as a result recruitment ‘chains’ are likely to break, which is very undesirable in RDS. The problem with having a low incentive, in turn, is that it is likely to create response bias: members of the target population who are better-off may not be incentivized enough. A high reward may be an ‘overkill’ for low-income members of the population, but it preempts creating this response bias.

A beneficial aspect of timing responses is that each question can be timed separately–not only the whole survey—which makes it possible to include specific questions in an otherwise suspect submission, or to discard a specific question in an apparently not suspect submission.

## Smartphone-based RDS

This section provides a descriptive account of the system developed by the authors for the purposes of an actual research project and explains why certain elements were introduced. The system was designed before the Covid-19 pandemic (in late 2019 / early 2020) but incidentally turned out to be in most respects well suited for restrictions on face-to-face contact.

Our motivation was to devise an automated digital system that would offer benefits of cost-reduction and fast data collection while minimizing the risk of fraud. We first considered employing a cross-platform solution, i.e. allowing participants to fill in the survey on any Internet-enabled device. We realized, though, that this would expose the study to a considerable risk of fraudulent use: most people own more than one Internet-enabled device and could fill in the survey multiple times (first legitimately and then illegitimately, using follow-up invitation codes sent to them). A cross-platform system could also encounter problems with distributing follow-up codes and rewards: they would need to be distributed by automatic email, which creates the risk of messages being blocked by spam filters. We therefore narrowed our attention to smartphones. Apart from allowing the use of online surveys, smartphones have built-in GPS, allowing for precise location reading. Crucially, unlike computers and tablets, they carry a unique ID in the form of the phone number.

In a nutshell, the system works as follows: the participant receives a unique invitation code from a ‘parent’ (usually by text, email or in person) and texts this code to a designated mobile number. Within a few seconds, the participant receives an automated text message with a link to the survey. The survey is filled online on the smartphone. Within a few seconds from completing the survey the respondent receives a text message with a reward (electronic shopping voucher) and another message with follow-up unique invitation codes for friends. When a friend completes the survey, the friend receives a primary reward, and a secondary reward is automatically texted to the ‘parent’.

The system comprises of three sub-systems interacting with each other:

### System A

This service is responsible for sending automatic text messages to participants. It needs to be a ‘programmable’ service, i.e. one that can be programmed using bespoke computer code. Several programmable text services available on the market accept code in PHP, Ruby, Python, Javascript, etc. We used Twilio (www.twilio.com/sms) and the program was written in Javascript.

### System B

This service is a database that consists of two tables, one with valid invitation codes and another one with rewards (electronic shopping vouchers, which take the form of a hyperlink). In our case the database was cloud-based (via Amazon AWS) but it could be hosted on an institutional server. S1 File describes those two tables in more detail.

### System C

This service is responsible for administering the online survey. There are several online survey services available on the market, but the chosen one needs to have three functionalities: (a) it needs to be able to retrieve information (respondent’s invitation code) from the survey URL; (b) it needs to be able to trigger the phone’s GPS functionality and to receive the reading; (c) it needs to have the ‘end URL’ option, whereby a custom URL link is triggered at the end of the survey. Additionally, two functionalities are technically not necessary but desirable: (d) setting a ‘prevent repeat participation’ cookie; (e) recording how long the respondent spent answering each question. We have used LimeSurvey (limesurvey.com), which offers all these functions.

Table 1 describes how the system works and how the user experiences it. We have decided to provide all details, as in our experience with digital RDS often ‘the devil is in the detail’.

**Table 1:** The chronological flowchart of user experience and system behaviour during data collection stage.

User experience	System behaviour					
The participant receives a unique invitation code from a ‘parent’ (via text / email / phone call / social media message, or in person), and a mobile phone number the code needs to be texted to.						
Participant texts his/her unique invitation code to the designated mobile phone number.						
	System A receives the text message. The content of the message is added to the log in system A, which can later be examined.					
	System A parses the message: blank characters are removed, and upper letters are converted to lower letters.					
	System A communicates with system B and checks whether the phone number from which the text was sent is already registered in the database.					
	Yes		No			
	Fraudulent attempt: the participant is trying to fill in the survey for a second time. System A sends a text to the participant ‘You have already participated in the survey’. No further action is taken.		The system checks if the content of the message is a valid invitation code (i.e. is in the database).			
			Yes			No
			The system checks if there is already a different phone number registered next to this invitation code (associated with it) in the database.			This is either a fraudulent attempt (the participant tried to guess the code) or the participant texted a valid code, but the message contained additional characters, e.g. ‘My code is abcd99’. System A sends a text to the participant ‘The code looks invalid. Please make sure you only include the code in your text, e.g. abcd99’.
			Yes	No		
			Fraudulent attempt: the participant is trying to use a code that has already been used by someone else. No further action is taken.	The system looks up if there is a phone number associated with the parent’s invitation code.		
				Yes	No	
				System B registers the participant’s phone number next to its invitation code (it associates the two). System A sends an automated text message to the participant with a welcome sentence and a link to the survey. The information about the participant’s invitation code is built into the survey link.	Fraudulent attempt: the participant has not been invited but has texted a correctly guessed code. No further action is taken.	
	All of the above operations take 3–5 seconds in total.					
The participant receives an automated text message with a welcome sentence and a link to the survey. In our case, the link looked like ‘https://i-sphere.limequery.com/165658?key=djtn36’, with the final 6 characters being the unique invitation code.						
The participant taps on the link in that text message.						
The survey opens in a web browser on the participant’s smartphone.						
	System C checks if the survey has been already completed on this smartphone / in this web browser (via a cookie).					
	Yes		No			
	Fraudulent attempt: the participant is trying to re-take the survey to claim another incentive. A message is shown to the respondent ‘You have already completed the survey’. No further action is taken.		System C registers the time the survey was started.			
			System C retrieves the participant’s invitation code from the survey link and stores it as a hidden variable.			
The participant reads a short study description on the first page of the questionnaire and is informed that more information is available upon tapping a link to the full project description. (It opens as a new web page). To progress, the participant needs to check the box saying ‘I have read the full study description and consent to take part’.						
The participant is taken to the next page of the questionnaire, where he/she is informed that the study needs to verify that the respondent is in the location where the survey is administered. The respondent is informed that upon clicking the ‘Next’ button, the smartphone is going to ask whether the respondent allows the survey to locate them geographically, and that he/she needs to agree to progress with the survey. The respondent is informed that the research team will only know the approximate location (+/- 1km), not the exact location.						
The respondent taps the ‘Next’ button on the smartphone.						
	System C asks the smartphone to read the location using GPS.					
	The smartphone sends the location coordinates to system C.		The smartphone does not send the location coordinates to system C.			
	System C registers the geographical coordinates, rounded up to provide approximate location, e.g. +/- 1km.		System C shows a message to the respondent: ‘It looks like ‘location services’ are not enabled on your phone. You need to go into the phone’s Settings = > Privacy = > Location Services and turn them on. Then tap again on the link to the survey’.			
	System C checks if the geographical coordinates lie within the area of the study.					
	Yes	No				
	The survey proceeds.	Fraudulent attempt: the participant is not present in the area under study. The survey is terminated and a message is shown to the respondent ‘Sorry, but it looks like you are not in [name of the town where the survey is conducted]’.				
The respondent continues to fill in the survey questionnaire.	System C registers how long the respondent spent answering each question.					
In the last question of the survey, the respondent is asked which supermarket he/she prefers (for the shopping voucher).						
The last page of the questionnaire displays a message informing the respondent that he/she can receive a further award for inviting friends to the survey, and that a text message with invitation codes is going to be sent immediately after completing the survey.						
Respondent taps the ‘Submit’ button at the end of the survey.						
	System C registers the time the survey was ended.					
	System C sets the cookie to ‘prevent repeat participation’.					
	System C communicates to System A the respondent’s invitation code and the name of the chosen supermarket. (Via the ‘End URL’ functionality).					
	System A communicates with system B to check if the invitation code is a valid one.					
	Yes			No		
	System A checks with system B whether there is a phone number associated with it.			Fraudulent attempt: the participant manually entered the survey URL with an invalid or empty code into the Internet browser. No further action is taken.		
	Yes		No			
	System A checks the value of the variable ‘survey_completed’ for this particular invitation code.		Fraudulent attempt: the participant manually entered the survey URL with a guessed (but valid) code into the Internet browser. No further action is taken.			
	The value is 0	The value is 1				
	System B changes the value of the variable ‘survey_completed’ from 0 to 1.	Fraudulent attempt: the participant manually entered the survey URL with an already used code into the Internet browser. No further action is taken.				
	System B looks up the first available (unused) shopping voucher for the supermarket of choice, of the desired value (primary reward–in our case, £10). System B changes the value of the variable ‘voucher_used’ from 0 to 1 for that particular voucher.					
	System A sends a text to the participant with the shopping voucher and an instruction that it needs to be shown to the cashier at the till.					
	System B looks up the parent’s mobile number and his/her supermarket of choice.					
	System B looks up the first available (unused) shopping voucher for the parent’s supermarket of choice, of the desired value (secondary reward–in our case, £5). System B changes the value of the variable ‘voucher_used’ from 0 to 1 for that particular voucher.					
	System A sends a text to the parent with the secondary reward and a message ‘Someone who you invited to the survey has just completed it. Below there is a link to the additional shopping voucher worth £5’.					
	System B retrieves follow-up invitation codes for whom the respondent is a ‘parent’ (in our case, three codes).					
	System A sends a text message to the respondent with the invitation codes and an instruction. (If there are no follow-up invitation codes in the database–i.e. the ‘chain’ is at its end—system A sends a text message saying ‘Sorry, the survey is now closed and therefore we are not sending further invitation codes’).					
	All of the above operations take 3–5 seconds in total.					
Respondent receives a text message with follow-up invitation codes and an instruction how to invite friends to the survey.						
Respondent distributes invitation codes among friends.						
	The survey reaches the target sample size.					
	Survey administrator deactivates the survey on system C, downloads survey responses from system C for analysis, and optionally exports a log of text messages from system A. Survey administrator sets system A to reply ‘Sorry, the survey is now closed’ to all incoming text messages. Survey administrator closes down system B.					
Participants who have received a link to the survey earlier and who visit the survey website are shown a message that the survey has been closed. Participants who text an unused invitation code to the designated mobile number are sent an automatic reply ‘Sorry, the survey is now closed’.						
	After a week or so the survey administrator closes down system A.					
Participants who text their unused invitation code to the designated mobile number do not receive any reply.						

The process outlined above needs to be complemented by two further actions at the data management stage. Survey submissions without the invitation code, or with an invalid invitation code, need to be discarded from the dataset. (Such submissions may occur if the respondent was trying to fraudulently guess the code by typing the survey URL directly into the Internet browser on a different device or in a different browser. Their submission would be received by system C). Additionally, if there are more than one survey submissions with an identical legitimate invitation code, only the first (earliest) completed instance is retained for analysis. This prevents fraud in the form of doing the survey two times after clearing the ‘prevent participation’ cookie, or by re-taking the survey on a different device by typing in the survey URL in the browser.

## Results and discussion

The data collection was stopped by the research team when the number of valid survey responses reached the target sample size of 300. The vast majority of survey responses came from ‘chains’ originating in two of the seven ‘seed’ respondents. One of these chains had 10 ‘waves’ and the other had 14 ‘waves’. This outcome was optimal in terms of RDS design; it is better if responses come from long chains originating in fewer seeds than short chains from multiple seeds.

In our real-life application the system performed well in terms of **fraud prevention**. This assertion is based on our examination of the log of messages coming to system A and the log of submissions from system C.

Starting with attempts at *multiple participation*, the system prevented fraud through safeguards built into systems A, B and C. With regards to systems A and B, 422 unique phone numbers interacted with these systems by sending a text message to our designated mobile number. Of these, 320 numbers can be categorized as non-fraudulent, i.e. they only sent one text with a valid, unused code. (Some of them obtained the link to the survey but did not click on the link, resulting in fewer than 320 survey responses). The scale of suspicious or fraudulent activity was therefore not marginal (102 out of 422), which underlines the importance of having safeguards in place. Of these 102, 80 unique numbers first texted a valid code but later texted another code (a used-up code, or one of the follow-up codes intended for ‘children’, or another code from the same ‘parent’). All these fraudulent attempts have been dealt with by systems A and B (see Table 1 for details).

It is not possible to know how many legitimate respondents to the survey tried to complete the survey for a second time (to claim another incentive) but were blocked by the ‘prevent repeat participation’ cookie. Only one legitimate respondent attempted to bypass this preventative mechanism and submitted two survey responses, either by clearing the ‘prevent repeat participation’ cookie or by entering the survey URL (with their valid code) on a different device / in a different browser. This attempt has been addressed at the data management stage, by keeping only the first response.

Moving on to the issue of *ineligible respondents*, of the 102 unique phone numbers that attempted suspicious activity 22 were ineligible respondents, i.e. they never texted a valid code to the system—they only texted an invalid code or a used-up code. These attempts have been dealt with by systems A and B.

Relatively few (5) respondents with a valid code started the survey but did not pass the geographical check. These fraudulent attempts have been dealt with by system C.

No-one tried to fill in the survey by manually entering the survey URL with an invalid, guessed code. However, there were 26 attempts at entering the survey URL manually with no code at all. These attempts have been dealt with at the data management stage, by discarding submissions without a valid code.

The system also performed well in terms of addressing the issue of poor **quality of responses**. System C (online survey) recorded both the total survey completion time and the completion time for each question, allowing for an informed evaluation of the extent to which poor quality was an issue. In our real-life case, the number of suspicious submissions was low. The median time to complete the survey was 12 minutes; three respondents (1%) took 3 minutes, six respondents (2%) took four minutes and a total of 26 respondents (14%) took six minutes or less. We retained these submissions but conducted a sensitivity analysis with and without them. Other researchers may decide that in their circumstances it is more appropriate to discard suspiciously quick responses. (To preserve the parent-child structure of RDS chains, such responses would need to be recoded to missing rather than completely deleted from the database).

In our view a key advantage of smartphone based RDS is the seamless, responsive and fast way that users experience this system: send a text with a code, immediately receive a text back with the link to the survey, take the survey on your phone, immediately receive a text with a shopping voucher and another text with follow-up codes for friends. There is no need to install any app or to have technological skills beyond the basic. A positive user experience is not just a ‘nice thing to have’ but is important for the success of the RDS: respondents are more likely to invite their contacts to participate in the survey if they had a positive experience themselves.

From a project design point of view, the attractiveness of smartphone based RDS lies in its low cost and short data collection period. The preparation of systems A and B took about 25 hours of IT specialist’s time, and our survey was active for one month before it reached the required sample size.

An obvious and important advantage of smartphone-based RDS data collection is that it does not require face-to-face contact, and as such it was optimal for social distancing restrictions during the Covid-19 pandemic. (The only aspect that was more difficult for the research team in comparison to non-pandemic times was recruiting ‘seed’ respondents. We had to rely on telephone / email contact for that, which is not as good for establishing the rapport as meeting in person).

Of particular importance in the context of a highly globalized world, the system can be made multilingual, with automated text messages showing in different languages depending on the language code assigned to a given invitation code, and the online survey automatically opening in the correct language. However, it needs to be remembered that language is one of the ‘population splitters’, so it may be more appropriate to conduct a few parallel RDS studies of different linguistic groups than one study of a linguistically heterogenous population.

The system has strengths in terms of data protection. In our application the respondent’s location was known to the precision of +/- 1km, which offers strong anonymity in an urban context. (The radius can be further increased, but that comes at a cost of including people who live outside of the area of study, i.e. ineligible participants). All three systems that we used were compliant with the General Data Protection Regulation (GDPR). The dataset with respondents’ phone numbers was stored on a different platform than the dataset of survey responses, which means that if someone accessed survey results in an unauthorized way, they would still not know the phone number of the respondent, rendering the survey data not ‘personal’.

### Limitations of smartphone based RDS

The system has some limitations. Starting with the more substantive and more difficult to overcome, there remain some sophisticated ways of exploiting the system to receive more than one incentive. To help the readers make their own opinion on whether these ways are easy or difficult to come up with by potential fraudsters–and by extension, whether fraud is likely to be common or uncommon–we invite the readers to pause reading at this point and think of how it might be possible to exploit the system.

- Filling in the survey first legitimately on own phone and then fraudulently on another person’s smartphone, using an invitation code designated for a ‘child’ / follow-up respondent. This scenario is perhaps not very likely if the fraudster tries to borrow a phone from an adult friend–people feel that a mobile phone is a highly private object and would not rush to pass one to another person to use–but is more likely in the case of families, with a parent using their child’s smartphone to commit the fraud. In our view this scenario requires quite a lot of ‘inventive thinking’ and as such should not be common.
- Filling in the survey first legitimately, then inserting a different SIM card into the smartphone (or switching to a second SIM on a dual-sim phone), and either clearing the cookie before re-taking the survey or doing the survey in a different browser (using an invitation code designated for a ‘child’ / follow-up respondent). This scenario requires some more advanced technological know-how from the fraudster. It can also be prevented by an advanced solution that occurred to us only with hindsight: hashing (anonymizing) the smartphone’s IMEI number ‘on the fly’ in system A and using it as a unique identifier, instead of the phone number. Unlike the phone number, IMEI does not change when a different SIM card is inserted into the phone.
- People who happen to own two smartphones–for example, one personal and one for work—can exploit this fact to commit fraud by filling in the survey on one phone and then again on another one, using a follow-up invitation code. However, it is perhaps less common these days to have a dedicated work phone than a few years ago (since current phone packages offer unlimited minutes and texts, there is no cost associated with making work-related phone calls and sending texts from a private mobile phone), and some work phones are ‘traditional’ keypad phones rather than smartphones.
- Someone who lives beyond the area under study but who happens to be present in that area (e.g. visiting, working) may take the survey while in the area. (Note however that system A can be set to ignore international numbers, so visitors from abroad would not be able to participate in the survey even if present in the study area).
- Demographic criteria for survey participation–such as being 18 or over, or being male–can be bypassed in the form of ineligible respondents taking part in the survey nevertheless. For this reason, we recommend that smartphone based RDS is used when demographic criteria are not needed. (One exception is when the study is about a specific migrant or ethnic minority group with their own language: having the survey questionnaire in that language acts as a filter in terms of eligibility. Our questionnaire was in Polish). Alternatively, it might be an option to not set any demographic requirements (e.g. any age instead of 18+), collect relevant demographic information in the survey questionnaire (respondent’s age), ask about the ‘network size’ in the target population (i.e. how many friends/acquaintances aged 18+ the respondent has), and recode responses from those not meeting the criteria (age < 18) to missing. Expert statistical advice would need to be sought, however, to find out if this workaround does not mislead the RDS estimator.

The remaining, less substantive limitations are as follows:

- There may not be an optimal point for the incentive level. If the incentive is low, RDS chains may ‘break’ too early, and additionally response bias may occur if better-off respondents are not incentivized enough. On the other hand, a high incentive may encourage potential fraudsters to think hard how to ‘play the system’.
- The issue of some potential respondents not having a smartphone will be decreasing in importance with time, as the saturation of smartphones approaches 100% of the adult population.
- In some developing countries or among extremely poor citizens in developed countries there may be an issue of potential respondents not having any mobile data. This can be addressed by employing a service that sends the user a small amount of mobile data for free, in exchange for watching an advert (e.g. www.opari.io).
- The researcher in charge of the system needs to have some IT skills to activate, monitor and deactivate it.

As far as we are aware, this has perhaps been the first-ever application of smartphone based, fully automated RDS. In our opinion it has delivered in terms of preventing common types of survey fraud and in terms of cost-effectiveness. Based on this, we believe that it could become RDS researchers’ preferred way of collecting data. This could be facilitated by the technological know-how and IT code being made available as freeware to the research community. It needs to be remembered, however, that RDS research that targets specific demographic groups is still likely to need to be face-to-face based, to prevent fraud from ineligible participants.

## Conclusions

The RDS is a method of surveying small or hard-to-reach populations that is designed to provide statistically sound results without the need to conduct a large survey of the population of which the target population is a sub-group. For all its usefulness, until recently the method has faced challenges around data collection, with researchers choosing between the traditional, face-to-face based data collection that was costly and lengthy and online-based data collection that was vulnerable to fraud. Smartphone based, fully automated RDS is a recent but promising methodological development that has prevented common types of survey fraud in a real-life application while being cost-efficient at the same time.

## Supporting information

S1 File This is a description of the two database tables forming system B. (PDF)
