# Supplementary material for: Smartphone-based Respondent Driven Sampling (RDS): A methodological advance in surveying small or ‘hard-to-reach’ populations
Source: PLoS One. 2022 Jul 21;17(7):e0270673. doi: 10.1371/journal.pone.0270673 (PMC9302716; doi:10.1371/journal.pone.0270673)
Supplement: S1 File — (PDF) [file pone.0270673.s001.pdf]

File S1: Tables forming the database in system B

System B was a database comprising two tables. The first table in the database held valid coupons (invitation codes) and phone numbers and had the following structure:

Table A1 The database table with valid coupons and phone numbers that was part of system B.

parent	child [a]	chain	recruit _n[b]	wave	invitation _token[c]	invited_ by[d]	phone_ number	survey_co mpleted	voucher _id[e]
null	1	1	null	0	djtn36	null		0	
null	2	2	null	0	tppb26	null		0	
null	3	3	null	0	fdsj44	null		0	
null	4	4	null	0	gswb59	null		0	
1	5	1	1	1	bckd94	djtn36		0	
1	6	1	2	1	gmce99	djtn36		0	
2	7	2	1	1	jpwa86	tppb26		0	
2	8	2	2	1	rrcn66	tppb26		0	
3	9	3	1	1	meab87	fdsj44		0	
3	10	3	2	1	nded46	fdsj44		0	
4	11	4	1	1	zjen83	gswb59		0	
4	12	4	2	1	rbdm45	gswb59		0	
…	…	…	…		…	…	…	…	…

[a] The first four ‘children’ in this table are ‘seed’ respondents

[b] Consecutive number of ‘children’ of a given ‘parent’

[c] Invitation code of the ‘child’

[d] Invitation code of the ‘parent’

[e] The unique ID of the primary reward voucher (see Table A2 below)

Note: our actual table had 15 ‘seeds’ and each ‘parent’ had three ‘children’. For presentational purposes the above table has four seeds and each parent has two children. The number of ‘waves’ in our table was 12 (with the wave for ‘seeds’ being 0). Coupons (invitation_token) were generated by us in Stata 15.

Columns parent-wave are technically not required but may be helpful in orientating oneself in the progress of data collection and for reducing the number of follow-up codes (see below).

Columns phone_number and voucher_id are empty at the start of data collection and get populated as the data collection progresses.

Note that child ID is unique.

The database that was used for our project allowed for dynamic, real-time modifications of tables (using SQL). We used this option to reduce the number of follow-up coupons from 3 to 2 past the 10th wave of recruitment and to add three extra waves to two chains that reached wave 12. Maximum care must be taken when modifying the table while data collection is active.

The second table in the database held the rewards (electronic shopping vouchers) and looked as follows:

Table A1 The database table with rewards (electronic shopping vouchers) that was part of system B.

id	vendor	url	value	voucher_used
1	ASDA	https://voucherexpress.vexrewards.com/reward/ 2abb8967602340f9b4266eae06ede618-10231515	10	0
2	ASDA	https://voucherexpress.vexrewards.com/reward/ 2436e3dfdde744ccb28cacd9005e7d52-10231656	5	0
3	Tesco	https://voucherexpress.vexrewards.com/reward/ 8cca935261954fa0a58f202354a699f3-10231475	10	0
4	Tesco	https://voucherexpress.vexrewards.com/reward/ 060984151a92468a892ea3c652f194ce-10231575	5	0
…	…	…	…	…

Note: you can copy the reward's URL and paste it into your web browser to preview it. These are not valid rewards. The number of supermarkets in our study was four.

The value from the column 'id' feeds into the column 'voucher_id' in the first table, as part of the process of delivering the primary reward to the respondent who completed the survey.
